# Prevalence of Cardiovascular Disease in Patients with Type 2 Diabetes Mellitus in Iran: A Systematic Review and Meta-Analysis

**DOI:** 10.1155/2020/3069867

**Published:** 2020-09-25

**Authors:** Mohsen Kazeminia, Nader Salari, Masoud Mohammadi

**Affiliations:** ^1^Department of Nursing, School of Nursing and Midwifery, Kermanshah University of Medical Sciences, Kermanshah, Iran; ^2^Department of Biostatistics, School of Health, Kermanshah University of Medical Sciences, Kermanshah, Iran

## Abstract

**Background:**

Type 2 diabetes mellitus (DM) is the most common type of DM and accounts for 90% of the cases. One of the most important complications of type 2 DM is cardiovascular complications, which are the most common cause of mortality in patients with DM. Various studies have reported different incidence rates of cardiovascular disease in patients with type 2 DM. However, no comprehensive review of previous studies has been done. This study is aimed at determining the prevalence of cardiovascular disease in patients with type 2 diabetes mellitus in Iran with a systematic review and meta-analysis.

**Methods:**

In this review, studies were first extracted searching domestic and international databases including SID, MagIran, IranMedex, IranDoc, Cochrane, Embase, ScienceDirect, Scopus, PubMed, and Web of Science (ISI), published between 2001 and September 2019. The random effects model was adopted for the analysis, and heterogeneity of the extracted studies was investigated with the *I*^2^ index. The data collected from the extracted studies were analyzed using a comprehensive meta-analysis (Version 2) software.

**Results:**

The prevalence of cardiovascular disease in patients with type 2 DM in Iran in 17 studies with a sample size of 9656 was 37.4% (95% CI: 31.4-43.8). Based on meta-regression, there was a significant difference on the effect of year of conducting the study and sample size with the prevalence of cardiovascular disease in patients with type 2 DM in Iran (*p* ≤ 0.001).

**Conclusion:**

The results of this study indicated that there was a high prevalence rate of cardiovascular disease in patients with type 2 DM in Iran. Therefore, appropriate strategies should be taken to improve this situation and trace and supervise it at all levels, providing feedback to hospitals.

## 1. Background

Type 2 diabetes mellitus (DM) is the most common type of DM and accounts for 90% of the cases. The prevalence of type 2 DM is steadily increasing [1], and its incidence in children has increased approximately tenfold [2]. It is estimated that there are currently 1.5 million patients with DM in Iran [3]. In 1997, the DM prevalence rate was about 125 million, and a recent World Health Organization (WHO) estimate shows that by 2025, the number of individuals with DM in the world will increase to 300 million [4]. Although the incidence rate of type 1 and type 2 DM is increasing worldwide, it is expected that the type 2 DM will increase more rapidly due to lifestyle changes leading to decreased physical activity and increased prevalence of obesity [5, 6].

Epidemiological studies have indicated that DM has a variable distribution in Iran. In a study conducted in Isfahan on subjects aged 35 years and older, the prevalence of DM was 7-8%, and this rate was 13.6% in Bushehr and 14.52% among individuals over 30 years in urban areas of Yazd province [7]. Type 2 DM is a familial disease, and there are convincing arguments in support of this claim. Genetic factors play an important role in the development of this disease. However, many of the underlying genes for DM are still unknown, but it is known to be polygenic and multifactorial. Various genetic loci are involved in the susceptibility of developing this disease. Environmental factors (such as nutrition and physical activity) also influence its phenotypic expression [5, 8].

The incidence of type 2 DM in identical twins is between 70% and 90%, and if one of them suffers from DM, the risk of the other twin developing diabetes is 50%. People with one parent with type 2 DM are at higher risk for developing DM; and obese individuals with type 2 diabetic parents are more likely to develop type 2 DM compared to those with parents without type 2 DM. In addition to the family history, other factors such as obesity, age, ethnicity, gestational DM, hypertension, and hyperlipidemia have been all involved in DM [9, 10].

Hypertension can be an early symptom of insulin resistance due to central obesity. A secondary hypothesis is that hypertension is a marker of endothelial dysfunction, which itself is a risk factor for insulin resistance, type 2 DM, and cardiovascular diseases (CVDs) [11]. Cardiovascular disease (CVD) is the name for the group of disorders of the heart and blood vessels and includes hypertension, coronary heart disease, stroke, peripheral vascular disease, heart failure, rheumatic heart disease, congenital heart disease, and cardiomyopathies. CVDs are the number one cause of death globally and more people die annually from CVDs than from any other cause, an estimated 17.3 million people died from CVDs in 2008, representing 30% of all global deaths [10–13].

Hypertension can be observed in 70% of patients with DM, and the risk of developing DM is 2 times higher in individuals with hypertension [12].

DM, especially the type 2, is often associated with lipid metabolism disorders. Increased plasma fatty acid levels play an essential role in increasing the insulin resistance. Additionally, plasma fatty acids cause dyslipidemia in DM by increasing low-density lipoprotein (LDL) and decreasing high-density lipoprotein (HDL). This androgenic function of lipoprotein (increased triglyceride, increased LDL, and decreased HDL) causes atherosclerosis and increased risk of CVDs, which is the most common cause of death in type 2 DM [13].

The incidence of coronary artery diseases (CADs) in individuals with type 2 DM is 2- to 4-fold higher than those without DM. The risk of myocardial infarction (MI) in patients with DM with no previous history of infarction appears to be as high as that of the individuals without DM with a history of MI [14]. The most common form of dyslipidemia in patients with type 2 DM is the elevated triglyceride levels and decreased HDL cholesterol [15].

The mean LDL cholesterol concentration in patients with type 2 DM is not significantly different from that in the individuals without DM. However, there may be qualitative changes in LDL cholesterol. In particular, patients with DM have smaller and denser LDL particles which make them more easily glycosylated and susceptible to oxidation and subsequently increase their risk of cardiovascular events [15–18].

According to a study conducted by Soltani and Fardin [19] in 2005, in the city of Isfahan located in north of Iran, 20.8% of patients with DM had ischemic heart disease [19]. In another relevant study by Abbasian et al. [20], 38% of patients with DM suffered from hypertension.

Given the effect of different factors on the prevalence of CVDs in patients with type 2 DM and lack of general/reliable statistics in this regard in Iran, we performed a comprehensive review of the literature published on patients in this geographical region and analyzed the results of these studies to assess the prevalence of CVDs in patients with type 2 DM in Iran.

This study is aimed at determining the prevalence of cardiovascular disease in patients with type 2 diabetes mellitus in Iran with a systematic review and meta-analysis. The findings of this study can be used to develop more precise planning to reduce CVDs in patients with type 2 DM.

## 2. Methods

In this systematic review and meta-analysis study, the prevalence rate of CVDs was evaluated in patients with type 2 DM in Iran without a time limit based on the studies published between 2001 and September 2019. To this end, the studies published in the Iranian databases including SID, MagIran, IranMedex, and IranDoc as well as the international databases Cochrane, Embase, ScienceDirect, Scopus, PubMed, and Web of Science (ISI) were searched with Persian keywords and their English equivalents including Prevalence, Complications, Cardiovascular, Diabetes, and Iran.

The observational (noninterventional) studies and all available full-text articles were included in this review. For more information, the references of the reviewed studies were also examined for access to other studies.

### 2.1. Selection of Studies

Initially, all studies referring to the prevalence of CVDs in patients with type 2 DM in Iran were collected and accepted by researchers (MK and MM) based on the inclusion and exclusion criteria. The exclusion criteria included unrelated cases, case reports, interventional studies, duplication of studies, unclear methodology, and inaccessibility of the full text of the study. In order to reduce bias, the studies were searched independently by two researchers (MK and MM), and in case of the lack of agreement on a study, it was judged by the third researcher (NS) or supervisor. A total of 24 studies entered into the third stage, i.e., the qualitative evaluation stage.

### 2.2. Qualitative Evaluation of Studies

The quality of the studies was evaluated based on the selected and related items of the 22-item STROBE checklist. Accordingly, the maximum quality score of 32 was considered, and papers with a score of less than 18 were considered to have low quality, and thus, they were excluded from the study [18]. In the present study, 17 high-quality and medium-quality studies were entered into the systematic meta-analysis review, and seven studies with a poor quality were excluded.

### 2.3. Data Extraction

All studies finally entered into the meta-analysis process were prepared for data extraction using a preprepared checklist. The checklist included the study title, the first author's name, year of the data collection, study location, sample size, prevalence of CVDs, and mean age.

### 2.4. Statistical Analysis

Since the prevalence rate had a binomial distribution, the prevalence variance was calculated using the binomial distribution variance formula and a weighted mean was applied to combine the prevalence rate of the different studies. In order to evaluate the heterogeneity of the selected studies, the *I*^2^ index test was used. In addition, the metaregression analysis was employed to investigate the relationship between the incidences of CVDs in patients with type 2 DM, the year of performing the study, and the sample size. In order to investigate publication bias, the Begg and Mazumdar test was used with a significance level 0.1 and its corresponding funnel plot. Furthermore, the sensitivity analysis was performed to evaluate the effect of each of the studies on the final result. The data was analyzed using the comprehensive meta-analysis implemented in “Version 2” software.

## 3. Results

**Figure 1:**
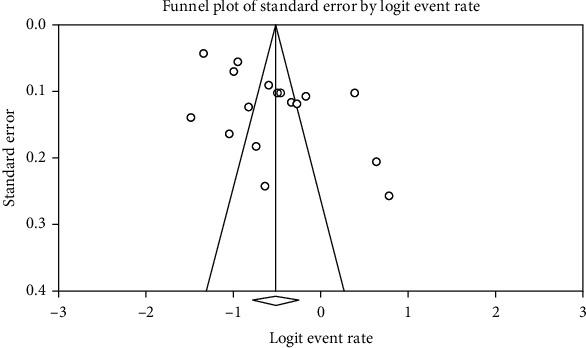
Funnel plot of results of the prevalence of cardiovascular disease in patients with type 2 diabetes mellitus (DM).

The probability of bias in the results by the funnel diagram and Begg and Mazumdar test at the significant level of 0.1 indicated that there is no bias in the present study (*p* = 0.174) (Figure 1).

**Figure 2:**
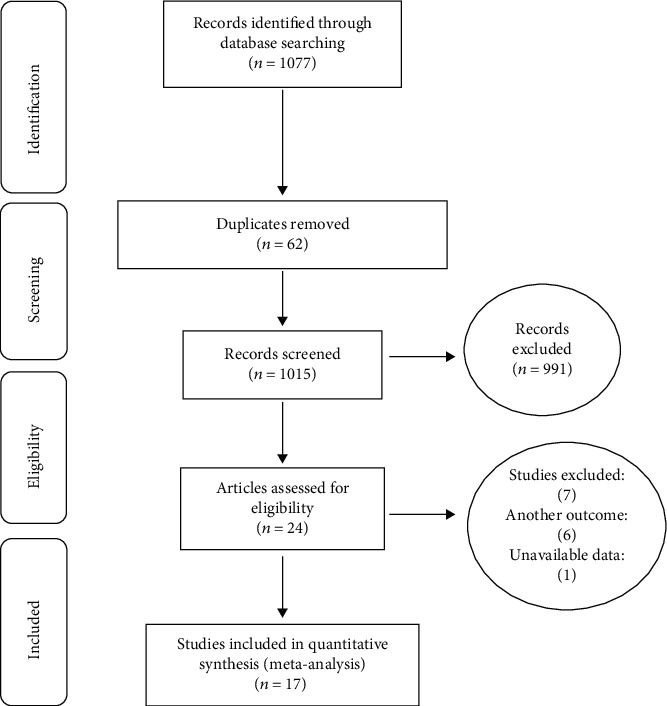
Flow diagram of study selection.

Based on PRISMA 2009, the studies published in the Iranian databases including SID, MagIran, IranMedex, and IranDoc and Cochrane, Embase, ScienceDirect, Scopus, PubMed, and Web of Science (ISI) were searched with Persian keywords and their English equivalents including Prevalence, Complications, Cardiovascular, Diabetes, and Iran between 2001 and September 2019. A total of 1077 articles were obtained. Subsequently, based on primary studies, after deleting 62 repetitive articles, there were 1015 articles with initial conditions to enter the study. Eventually, 17 articles were included in the meta-analysis process after secondary study with deletion of 991 unrelated articles and 7 articles which abstracts and full texts were unavailable and their quality was low (Figure 2).

The search terms were as follows: (((((Cardiovascular Diseases [Title/Abstract]) OR CARDIOVASC DIS [Title/Abstract]) OR CVD [Title/Abstract] AND Blood glucose [Title/Abstract]) OR Hyperglycemia [Title/Abstract]) AND Diabetes [Title/Abstract]) OR Non-insulin dependent diabetes [Title/Abstract]) AND Nephropathy) OR Diabetic Nephropathies))))).

**Figure 3:**
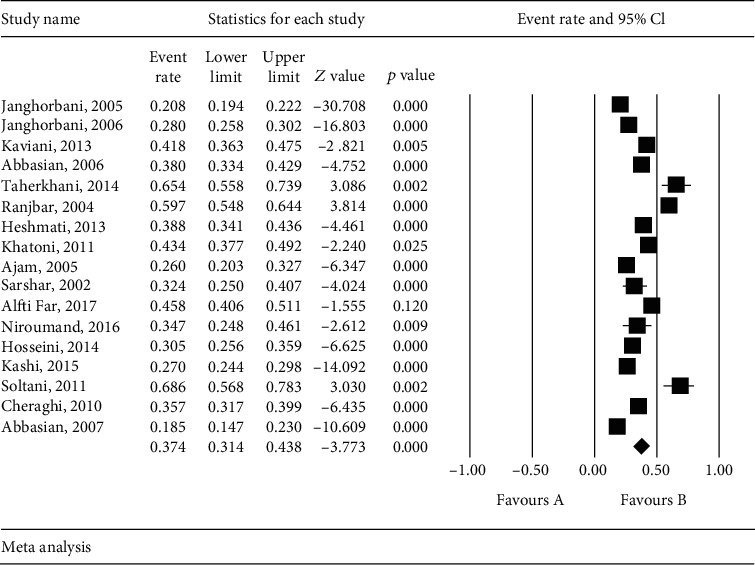
Prevalence of cardiovascular disease in patients with type 2 diabetes mellitus (DM) and 95% confidence interval in Iran. The middle point of each line shows the prevalence of cardiovascular disease in each study, and the rhombic figure shows the prevalence of cardiovascular disease in patients with type 2 DM in Iran for the whole studies.

Based on the results of the heterogeneity of the studies (*I*^2^ = 96.8) and due to the heterogeneity of the selected studies, the random effects model was conducted to combine the studies and the joint prevalence estimation. The total sample size was 9656 individuals with the mean age of subjects in each study presented in Table 1. The lowest and the highest sample sizes were related to Soltani et al. (2011) and Janghorbani et al. (2005) with 70 and 3202 subjects, respectively; and the highest and lowest prevalence of CVDs in patients with type 2 DM in Iran were, respectively, related to the studies by Soltani and Fardin Far [19] and Abbasian et al. [20] (Table 1). According to the meta-analysis, the prevalence of CVDs in patients with type 2 DM in Iran was estimated to be 37.4% (95% CI: 31.4-43.8%) (Figure 3).

**Figure 4:**
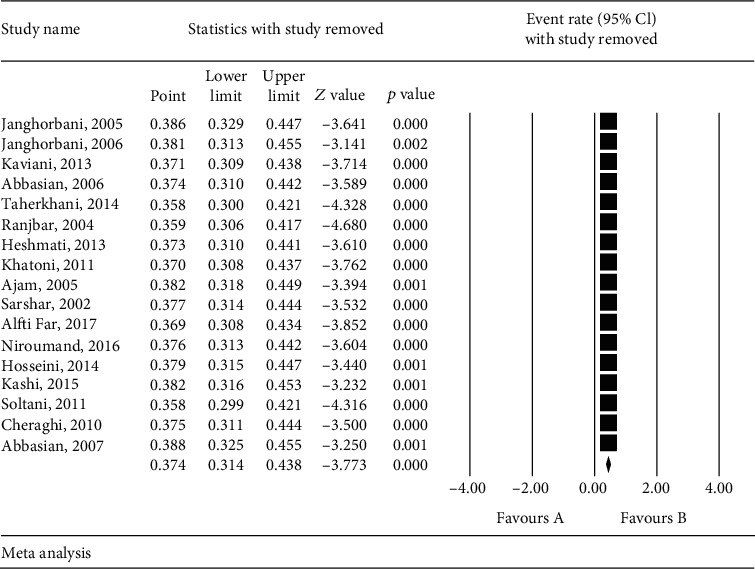
Sensitivity analysis results.

The sensitivity analysis was performed in accordance with Figure 4 to ensure the stability of the study results; after removing each study, results did not change (Figure 4).

**Figure 5:**
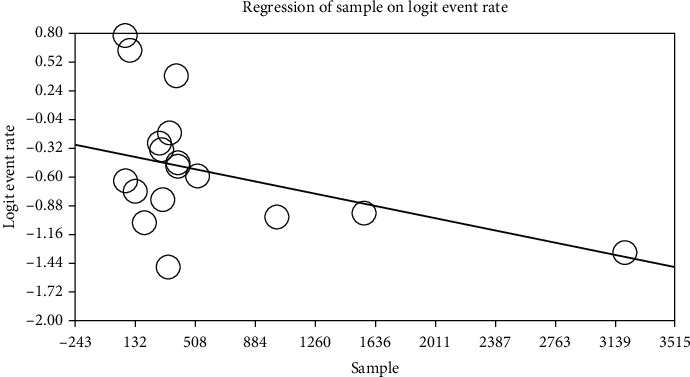
Metaregression of the relationship between the sample size and prevalence of cardiovascular disease in patients with type 2 diabetes mellitus (DM) in Iran.

**Figure 6:**
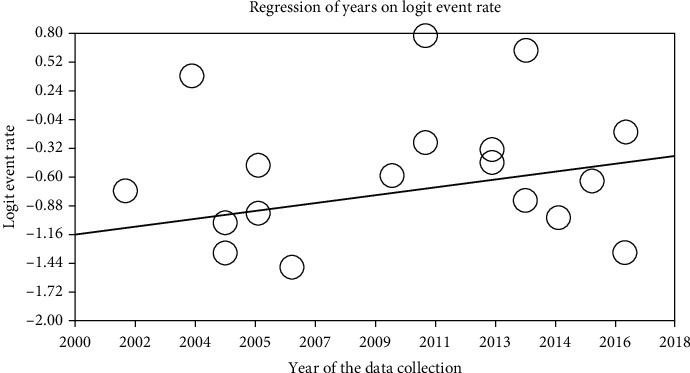
Metaregression of the relationship between the year of study and prevalence of cardiovascular disease in patients with type 2 diabetes mellitus (DM) in Iran.

The relationship between the year of conducting the study (*p* ≤ 0.001) and the sample size (*p* ≤ 0.001) with the prevalence of cardiovascular disease in patients with type 2 DM in Iran was investigated using the metaregression. Significant differences were observed between cardiovascular disease and the two above cases. The prevalence of cardiovascular disease in patients with type 2 DM in Iran was increased with the increase in the year of conducting the study and decreased with the increase in the sample size (Figures 5 and 6).

**Figure 7:**
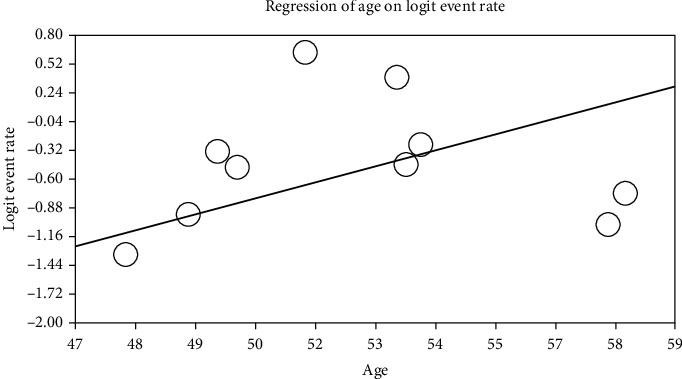
Metaregression of the relationship between the age of participants in the study and prevalence of cardiovascular disease in patients with type 2 diabetes mellitus (DM) in Iran.

With increasing age of participants in the study, the prevalence of cardiovascular disease in patients with type 2 DM in Iran increases, which is statistically significant (*p* ≤ 0.001) (Figure 7).

## 4. Discussion

The aim of this study was to determine the prevalence of cardiovascular disease in patients with type 2 DM in Iran. DM is one of the most common diseases worldwide. The American Diabetes Association (ADA) reported that in 2007, $174 billion was spent treating patients with DM, of which $58 billion was spent to mitigate the damages due to the long-term complications of this disease [34]. This disease has an increasing trend and has been predicted to rise from 285 million in 2010 to 439 million in 2030 [36].

In the present study, the prevalence of cardiovascular disease in patients with type 2 DM in Iran was 37.4%. In the study performed by Liu et al. in China, the prevalence of CVDs in patients with type 2 DM was 30.1% [37]. In addition, in a study conducted by Shi et al. on morbidity associated with chronic complications of DM in China, CVDs were the most common chronic complication of type 2 DM [38]. The rate of cardiovascular disease in patients with type 2 DM was 26% in South Korea [39]. The results of a study in Denmark in 2010 reported a complication rate of 32 to 40% [40]. Patients with DM undergo periodic evaluation of renal and ocular complications; however, there is no specific plan to assess the related cardiovascular programs. Given the high prevalence of cardiovascular disease in patients with DM and lack of a clear plan for evaluating these complications, it is recommended that CVDs should be prevented by taking preventive measures such as regular exercise and developing cardiovascular periodic evaluation programs.

The long life and quality of life of patients with DM depend on the progression and severity of chronic complications, especially CVDs [41, 42]. The high prevalence of complications in these patients is a serious issue as these complications are not reversible and could cause damage to the organs and result in serious health problems for the patients. This could also incapacitate the patients and consequently incur heavy medical cost burden on the patients and society.

Grobbee showed that CVDs and hypertension are common problems in patients with DM; besides, obesity is one of the predisposing factors for CVDs [43]. In a report by the WHO published in 2010, obesity and overweight have been identified as the most important contributors to the rising trend of type 2 DM [44]. Moreover, numerous other studies have also suggested obesity to be the most important risk factor for type 2 DM [45–47]. Therefore, it seems that by the continuous control of the blood lipids and preventive measures to stop its increasing rate, the prevalence of type 2 DM and hence its chronic complications, especially cardiovascular disease, can be reduced in society. Adequate training on obesity and on reducing its complications is needed to be provided.

The incidence of DM and obesity has rapidly increased in the last century, and the morbidity and mortality resulting from these two epidemics have caused enormous health problems for human societies [48–51]. Type 2 DM, which is the most common type of DM, could be developed due to the presence of an inherited background as well as environmental factors as the most affecting factors [52]. In many cases, the lack of a healthy nutrition and immobility would first cause prediabetes and then diabetes emerges [53].

According to a systematic review and meta-analysis reported in Ray et al., out of 1497 cases of nonlethal myocardial infarction, 2318 cases of cardiovascular diseases, 1127 cases of stroke, and 2892 deaths, it was found that glycemic control resulted in a 17% decrease in the incidence of nonlethal MI and a 15% decrease in CADs, but with no significant effect on stroke and mortality among patients [54]. Nutrition, physical activity, glycemic control, and training of patients are the basis of DM treatment for all diabetic patients. Medical treatment should be accompanied by nutritional therapy and physical activity, in addition to considering weight loss and healthy lifestyle when choosing a suitable treatment [55, 56].

The findings in this study indicated that there is a high incidence of cardiovascular disease in patients with type 2 DM in the population under study. As a result, interventions must be performed in lifestyle changes as well as regular control of blood pressure, cholesterol, and blood glucose in patients to prevent the disease and reduce DM-related complications. Since cardiovascular diseases in patients with DM are primarily preventable and can be controlled and treated in case of developing the complication, patients with DM need thus to be fully trained about this disease and learn the ways to prevent it. Moreover, the complications can be controlled and treated with early and timely diagnosis.

Given the high prevalence of cardiovascular disease in patients with type 2 DM in Iran, it is suggested that physicians pay more attention to the symptoms of this disease and that media training should be carried out with the aim to raise the awareness of individuals to reduce the delay in diagnosis. Studies are also recommended to be carried out on the prevalence of cardiovascular disease in patients with type 2 DM in other parts of the world to find out the worldwide rate.

## 5. Conclusion

The results of this review revealed that there is a high prevalence rate of cardiovascular disease in patients with type 2 DM in Iran. Therefore, appropriate strategies should be taken to improve this situation and trace and supervise it at all levels, providing feedback to hospitals.

## Figures and Tables

**Table 1:** Characteristics of included studies in the prevalence of cardiovascular disease in patients with type 2 diabetes.

Author [reference]	Year of the data collection	Mean age (years)	City	Sample size	Prevalence (%)	Quality
Janghorbani and Amini [21]	2005	48.3	Isfahan	3202	20.8	High
Abbasian et al. [22]	2006	49.6	Shahroud	400	38.0	High
Abbasian and Delorian [20]	2007	50.2	Shahroud	340	18.5	High
Janghorbani et al. [23]	2006	50.6	Isfahan	1566	28.0	High
Sarshar and Chamanzari [24]	2002	52	Gonabad	136	32.4	High
Hosseini et al. [25]	2014	53.9	Tehran	305	30.5	High
Taherkhani and Safi [26]	2014	54.09	Tehran	104	65.4	High
Kashi et al. [27]	2015	54.4	Sari	1021	27.0	High
Khatoni et al. [28]	2011	58.25	Qasr-e Shirin	286	43.4	High
Kaviani et al. [29]	2013	58.6	Khorramabad	299	41.8	High
Ranjbar et al. [30]	2004	—	Shiraz	392	59.7	Medium
Heshmati et al. [31]	2013	—	Fereydunkenar	400	38.8	High
Ajam et al. [32]	2005	—	Gonabad	347	26.0	Medium
Alfti Far et al. [33]	2017	—	Hamadan	89	45.8	High
Niroumand et al. [34]	2016	—	Northeast of Iran	75	34.7	High
Soltani and Fardin Far [19]	2011	—	Birjand	70	68.6	Medium
Cheraghi et al. [35]	2010	—	Shadegan	521	35.7	Medium

## Abbreviations

DM: Diabetes mellitus
CVDs: Cardiovascular diseases
WHO: World Health Organization
HDL: High-density lipoprotein
LDL: Low-density lipoprotein
STROBE: Strengthening the reporting of observational studies in epidemiology for cross-sectional study
PRISMA: Preferred reporting items for systematic reviews and meta-analysis.

## References

[1] Boyle J. P., Honeycutt A. A., Narayan K. M. V. (2001). Projection of diabetes burden through 2050:Impactofchanging demography and disease prevalence in the U.S. *Diabetes Care*.

[2] Lean M., McCombie L., McSorely J. (2019). Trends in type 2 diabetes. *BMJ*.

[3] Larijani F., Zahedi F., Aghakhani S. (2003). Epidemiology of diabetes mellitus in Iran. *Shiraz E-Medical Journal*.

[4] American Diabetes Association (2017). Prevention or delay of type 2 diabetes: standards of medical care in diabetes-2018. *Diabetes Care*.

[5] Powers A., Braunwald E., Fauci A. S., Kasper D. L. (2001). Diabetes mellitus. *Harrison's principles of internal medicine*.

[6] King H., Aubert R. E., Herman W. H. (1998). Global burden of diabetes, 1995–2025: prevalence, numerical estimates, and projections. *Diabetes Care*.

[7] Afkhami-Ardekani M., Vahidi S., Vahidi A., Ahmadia M. H. (2001). Epidemiological survery of NIDDM in persons over 30 years old in Yazd province. *Journal of Shaheed Sadoughi University of Medical Sciences*.

[8] Levy J. C., Aetiology A., Hitman G. (1999). *Type 2 diabetes prediction and prevention*.

[9] Bjørnholt J. V., Erikssen G., Liestøl K., Jervell J., Thaulow E., Erikssen J. (2000). Type 2 diabetes and maternal family history: an impact beyond slow glucose removal rate and fasting hyperglycemia in low-risk individuals? Results from 22.5 years of follow-up of healthy nondiabetic men. *Diabetes Care*.

[10] Bennett P. H., LeRoith D., Talor S. I., Olefsky J. M. (2004). Epidemiology of diabetes mellitus. *Diabetes Mellitus: A Fundamental and Clinical Text*.

[11] Tooke J., Goh K. (1999). Vascular function in Type 2 diabetes mellitus and pre-diabetes: the case for intrinsic endotheliopathy. *Diabetic Medicine*.

[12] Hill Golden S., Wang N.-Y. J., Klag M. A., Meoni L. L., Brancati F. (2003). Blood pressure in young adulthood and the risk of type 2 diabetes in middle age. *Diabetes Care*.

[13] Steinmetz A. (2003). Treatment of diabetic Dyslipoproteinemia. *Experimental and clinical endocrinology & diabetes*.

[14] Evans M., Khan N., Rees A. (1999). Diabetic dyslipidaemia and coronary heart disease: new perspectives. *Current Opinion in Lipidology*.

[15] American Diabetes Association (2004). Dyslipidemia management in adults with diabetes. *Diabetes Care*.

[16] Agashe S., Petak S. (2018). Cardiac Autonomic Neuropathy in Diabetes Mellitus. *Methodist Debakey Cardiovasc J*.

[17] Lejay A., Fang F., John R. (2016). Ischemia reperfusion injury, ischemic conditioning and diabetes mellitus. *J Mol Cell Cardiol*.

[18] Vandenbroucke J. P., von Elm E., Altman D. G. (2007). Strengthening the reporting of observational studies in epidemiology (STROBE). *Epidemiology*.

[19] Soltani M., Fardin F. S. (2011). *Relative frequency of diabetes complications in diabetic patients admitted to internal ward of Birjand Imam Reza and Vali Asr Hospital from October 2000 to October 2001, Birjand University of Medical Sciences. Numbe 2*.

[20] Abbasian M., Delorian Z. M. (2007). Prevalence of diabetes complications in patients referred to Shahroud Diabetes Clinic. *Journal of Knowledge and Health*.

[21] Janghorbani M., Amini M. (2005). Hypertension in type 2 diabetes mellitus in Isfahan, Iran: incidence and risk factors. *Diabetes research and clinical practice*.

[22] Abbasian M., Delorian Zadeh M., Hosein Zadeh S., Norozi P. (2006). *Prevalence of diabetes complications in patients referred to Shahroud Diabetes Clinic, 9th Iranian Congress of Nutrition*.

[23] Janghorbani M., Amini M., Tavassoli A. (2006). Coronary heart disease in type 2 diabetes melittus in Isfahan, Iran. *Acta cardiologica*.

[24] Sarshar N., Chamanzari H. (2002). Diabetes complications in patients referred to Diabetes Clinic of Gonabad. *Journal of Gonabad University of Medical Science*.

[25] Hosseini M. S., Rostami Z., Saadat A., Saadatmand S. M., Naeimi E. (2014). Anemia and microvascular complications in patients with type 2 diabetes mellitus. *Nephro-urology monthly*.

[26] Taherkhani M., Safi M. (2014). Prevalence of microvascular complications and coronary artery disease in patients with type 2 diabetes. *Journal of Research in Medical School, Shahid Beheshti University of Medical Sciences*.

[27] Kashi Z., Bahar A., Akha O. (2015). Ischemic heart disease and related factors in patients with diabetes mellitus type II. *Journal of Mazandaran University of Medical Sciences*.

[28] Khatoni A. R., Abdi A. R., Hosseini E., Fatahpor T., Faizi H. (2011). Risk factors and complications of type 2 diabetes in Qasr Shirin Diabetes Center in the year 2010. *Journal of Vulnerable Groups Nursing, Boshehr University of Medical Sciences*.

[29] Kaviani M., Abdolahian M., Almaci V., Anbari K., Jefferiesteh A. (2013). *Frequency of chronic complications of type 1 diabetes in patients candidate for insulin use due to hyperglycemia, Lorestan University of Medical Sciences. the period 15, Numbe 4*.

[30] Ranjbar Gh H., Soeid M., Rajaei H., Sadegh A. S. (2004). The incidence of chronic complications of diabetes in patients referred to clinics affiliated to Shiraz University of Medical Sciences during a 12 year period. *Iranian Journal of Diabetes and Lipid*.

[31] Heshmati H., Behnam Por N., Khorasani F., Moghadam Z. (2013). Prevalence of chronic diabetes complications and some related factors in type 2 diabetic patients referred to Diabetes Center of Fereidoonker. *Journal of Neyshabour School of Medical Sciences*.

[32] Ajam M., Raihani T., Mirsani A. A., Nazemi S. H. (2005). *Evaluation of chronic physical complications of diabetic patients referred to Gonabad Hospital, Gonabad University of Medical Sciences. the period 11, Numbe 4*.

[33] Alfti Far M., Karami M., Shokri P., Hosseini S. M. (2017). Prevalence of diabetes mellitus complications and associated risk factors in patients referred to Hamadan diabetes center. *Scientific Journal of Hamadan Nursing & Midwifery Faculty*.

[34] Niroumand S., Dadgarmoghaddam M., Eghbali B. (2016). Cardiovascular disease risk factors profile in individuals with diabetes compared with non-diabetic subjects in north-east of Iran. *Iranian Red Crescent Medical Journal*.

[35] Cheraghi Z., Amori N., Dosti A., Bitaraf A. (2010). *Prevalence of type 2 diabetes complications in diabetic patients covered by diabetes unit of Shadegan Health Center in 2009, Journal of Knowledge and Health, Special Issue of the 6th Iranian Epidemiology Congress. the period 5*.

[36] Ogurtsova K., da Rocha Fernandes J. D., Huang Y. (2017). IDF Diabetes Atlas: Global estimates for the prevalence of diabetes for 2015 and 2040. *Diabetes Research and Clinical Practice*.

[37] Liu Z., Fu C., Wang W., Xu B. (2010). Prevalence of chronic complications of type 2 diabetes mellitus in outpatients-a cross-sectional hospital based survey in urban China. *Health and Quality of Life Outcomes*.

[38] Shi W., Li X., Li J. (2004). The morbidity of chronic diabetic complication with logistic regression analysis of related potential risk factors. *Zhonghua liu xing bing xue za zhi= Zhonghua liuxingbingxue zazhi*.

[39] Moon S.-S., Choi Y.-K., Seo H.-A. (2010). Relationship between cardiovascular autonomic neuropathy and coronary artery calcification in patients with type 2 diabetes. *Endocrine Journal*.

[40] Poulsen M. K., Henriksen J. E., Dahl J. (2010). Left ventricular diastolic function in type 2 diabetes mellitus: prevalence and association with myocardial and vascular disease. *Circulation: Cardiovascular Imaging*.

[41] Krolewski A. S., Kosinski E. J., Warram J. H. (1987). Magnitude and determinants of coronary artery disease in juvenile-onset, insulin-dependent diabetes mellitus. *The American Journal of Cardiology*.

[42] Delavari A. R., Mahdavi Hezavei A. R., Norozi Nezhad A., Yarahmadi S. H. (2003). *National Program of Diabetes Prevention and Control*.

[43] Grobbee D. E. (2003). How to ADVANCE prevention of cardiovascular complications in type 2 diabetes. *Metabolism*.

[44] Rodríguez A., Delgado-Cohen H., Reviriego J., Serrano-Ríos M. (2011). Risk factors associated with metabolic syndrome in type 2 diabetes mellitus patients according to World Health Organization, Third Report National Cholesterol Education Program, and International Diabetes Federation definitions. *Diabetes, metabolic syndrome and obesity: targets and therapy*.

[45] Sanada H., Yokokawa H., Yoneda M. (2012). High body mass index is an important risk factor for the development of type 2 diabetes. *Internal Medicine*.

[46] Hadaegh F., Zabetian A., Harati H., Azizi F. (2007). The prospective association of general and central obesity variables with incident type 2 diabetes in adults, Tehran lipid and glucose study. *Diabetes Research and Clinical Practice*.

[47] Kawahara R., Amemiya T., Yoshino M., Komori T., Shibata N., Hirata Y. (1990). Adverse effects of obesity on lipid and lipoprotein levels in the patients with non-insulin dependent diabetes in the young. *Diabetes Research and Clinical Practice*.

[48] Anderson R. J., Freedland K. E., Clouse R. E., Lustman P. J. (2001). The prevalence of comorbid depression in adults with diabetes: a meta-analysis. *Diabetes Care*.

[49] De Groot M., Anderson R., Freedland K. E., Clouse R. E., Lustman P. J. (2001). Association of depression and diabetes complications: a meta-analysis. *Psychosomatic Medicine*.

[50] Eren I., Erdi Ö., Şahin M. (2008). The effect of depression on quality of life of patients with type II diabetes mellitus. *Depression and Anxiety*.

[51] World Health Organization Diabtes http://www.who.int/mediacentre/factsheets/fs312/en.

[52] Alberti K. G. M. M., Zimmet P., Shaw J. (2007). International Diabetes Federation: a consensus on type 2 diabetes prevention. *Diabetic Medicine*.

[53] International Diabetes Federation (2012). *IDF Diabetes Atlas*.

[54] Ray K. K., Seshasai S. R. K., Wijesuriya S. (2009). Effect of intensive control of glucose on cardiovascular outcomes and death in patients with diabetes mellitus: a meta-analysis of randomised controlled trials. *The Lancet*.

[55] Joslin Diabetes Center (2007). *Clinical guideline for pharamacological of type 2 diabetes*.

[56] American Diabetes Association (2013). Economic costs of diabetes in the U.S. in 2012. *Diabetes Care*.

